# Cognitivism and psychomotor skills in surgical training: from theory to practice

**DOI:** 10.5116/ijme.5b9a.129b

**Published:** 2018-09-28

**Authors:** David Shaker

**Affiliations:** 1 The University of Queensland, Rural Clinical School, Rockhampton, Australia

**Keywords:** Cognitivism, psychomotor skills, surgical training, theory to practice, Australia

## Introduction

Learning theories are essential to developing scientifically sound educational methods. However, they are frequently viewed as being of merely academic interest. This article aims to offer some perspective on translating learning theory into teaching practice. It looks at the process of learning psycho-motor skills from a cognitive perspective and how to translate this into surgical training practice. I will start by discussing the cognitive aspects of learning psycho-motor skills. This will be followed by extrapolating an applicable principle; I will then describe an example of applying cognitivism to teaching psychomotor skills in surgical training.

### Cognitive theory and acquisition of psychomotor skills

Cognitivism as a learning theory is concerned with the thought process associated with learning. While the role of cognitive process is apparent in the cognitive domain, it is less so in psycho-motor or affective domains.

Using Fitts and Posner model of human performance,^1^ Hamdorf and Hall described the acquisition of surgical skill as proceeding in three stages; cognition, integration and automation.^2^ In the cognitive stage, the learner intellectualizes the task and executes it. This proceeds to integration when the learner refines the technique for the most efficient use of movements. Achieving automation signifies the individual can perform the task with minimal cognitive input. This results from what is called “hot wiring of a map of the whole performance in the brain”.^2^

The cornerstone of Cognitivism is identified to be, the memory and processing of information. Young and colleagues summarise Atkinson and Shriffin’s model of human memory. They describe three stages, sensory register, working memory and long-term memory.^3^ The sensory register allows entry of information. While large amounts of data are processed, only a small fraction passes to short-term memory to be arranged in chunks and schemata and stored in the long-term memory.^3^ John Sweller, using this model, suggested that increased cognitive load beyond the limited capacity of the short-term memory negatively affects learning. This is known as Cognitive Load Theory.^4^ While cognitive load theory is the most commonly quoted aspect of cognitivism, it represents one aspect of a broader learning theory.

In considering the role of the cognitive process in the acquisition of psychomotor skill, five components can be identified. These include Attention, Perception, Concept formation, Memory and Learning.^5^

As the learner approaches a psychomotor task, a large amount of visual, auditory, haptic and other stimuli are gathered. Through the process of attention, data is filtered, allowing only a small relevant fraction to pass to short-term memory, minimising cognitive overloading. Meanwhile, transfer of appropriate stimuli to short-term memory is considered essential in creating internal image and representation of body and space, leading to “Perception” and “Conceptualisation”. It is this “Concept Formation” which allows storage of the task into the long-term memory. At this stage, the learner is ready to recall the process and execute the task again if needed.^5^

Once this process is achieved, the learner will progress to refine the execution most efficiently and then; further practice will facilitate hot-wiring map formation in the brain, to progress the learner to automation.

One can argue that progress from the cognitive stage to the integrative stage depends on the ability to conceptualise the components of the skill, which in turn reflects the efficiency of attention. Suboptimal filtration will result in cognitive overload while inappropriate excessive filtration of stimuli will hamper the creation of accurate internal imaging of body and space.

### From theory to practice; an applicable principle

To help to translate the theory into practice, a principle is extrapolated from the theory and applied in teaching and learning of psychomotor skills.

Most work done on the cognitive aspects of learning surgical skills have concentrated on the role of “cognitive load theory”, aiming to minimise exogenous cognitive load and gradually build up the intrinsic load.^6^ However, there are other components in the cognitive model of learning psycho-motor skill which can be targeted in teaching and training. From the above discussion, it is clear that the tremendous amount of sensory stimuli accessing the sensory register of the trainee during learning a surgical procedure can be refined into what is beneficial and what is not, in what I call “Attention Refinement”. Attention refinement can be defined as attracting the attention to further away but significant, parts of the field (Significant blind spots), and detracting the attention from insignificant parts in the direct visual field (insignificant detractors), at the same time attracting the attention to the role of spatial relation of the instruments and body movements (external factors) in facilitating or hindering the task execution.

I suggest that in the process of teaching a surgical skill, attention refinement will shorten the cognitive stage of learning and facilitate the transition to integration.

### An example of the principle in action

Using the above principle, an instructional session was designed for 4th-year trainee in Obstetrics and Gynaecology in relation to laparoscopic salpingectomy for ectopic pregnancy. The trainee had already been through the first three of Peyton’s Four Steps^7^. The session was delivered before a planned operating session, where the trainee was expected to perform the procedure under supervision.

A non-edited video, of the procedure previously performed by the supervisor, was played. The video was divided into three parts, and the trainee was asked to document what could be seen in the field and its significance. Then the supervisor discussed the significant and insignificant aspects of the field in terms of progressing safely with the procedure. The supervisor then brought to attention some significant blind spots which were not noted by the trainee.

Examples of insignificant aspects that attracted trainee’s attention were bowel adhesions. The supervisor pointed out that while the ectopic to be removed was on the right side; adherent bowel was on the left side has no implications on current procedure. On the other hand, the trainee failed to bring to attention that the ectopic pregnancy was adherent to the pelvic sidewall just over the course of the ureter and needed to be released before using diathermy to remove the tube. These are only two of many potential points.

The trainee progressed to perform the procedure under direct supervision. After finishing the procedure, the trainee was invited to discuss what were the significant blind spots and insignificant detractors during the procedure.

While the central core of surgical instructions falls within the “four steps” as described by Peyton^7^ the example described, focuses on different aspects, namely desirable and undesirable attention to stimuli in the form of visual stimuli from existing anatomical structures.

## Conclusions

While cognitivism is essentially a theoretical framework to understand the process of learning, it is applicable in day to day teaching. This is achieved by extracting a key principle from the concept and applying it to teaching. The article described one example of “attention refinement” applied to optimise surgical training instructions and facilitate the acquisition of surgical skills, in this case, laparoscopic procedure. However, there are many potential applicable principles extractable from the theory. This approach, if adopted, will have wide implications in teaching and learning surgical skills. By considering teaching practices which promote any of the five components of the cognitive model, one can influence the process.

### Acknowledgement

The author would like to thank Mrs. Angela Cameron-Tomkinson for her help with manuscript formatting.

### Conflict of interest

The author declares that he has no conflict of interest
